# Supplementary material for: The PROVIT Study—Effects of Multispecies Probiotic Add-on Treatment on Metabolomics in Major Depressive Disorder—A Randomized, Placebo-Controlled Trial
Source: Metabolites. 2022 Aug 21;12(8):770. doi: 10.3390/metabo12080770 (PMC9414726; doi:10.3390/metabo12080770)
Supplement: Supplementary file 1 [file metabolites-12-00770-s001.zip › metabolites-1857730-supplementary.pdf]

Article - Supplementary

The PROVIT study - Effects of a multispecies probiotic on metabolomics in major depressive disorder a randomized, placebo-controlled trial

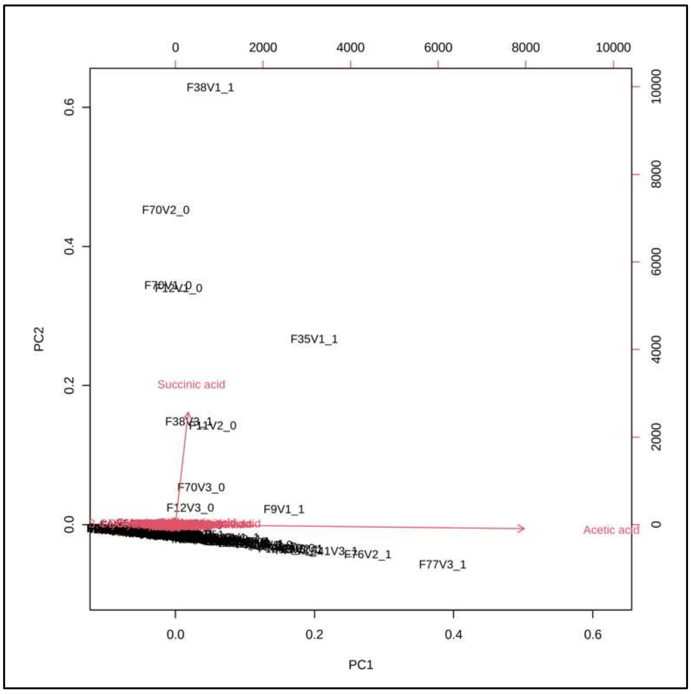

Figure S1. Untargeted metabolomics of placebo and probiotics group in stool. Principle component analysis (PCA) biplot analysis strong variations of metabolite concentrations, with PC1 being dominated by acetate and PC2 being dominated by succinate.

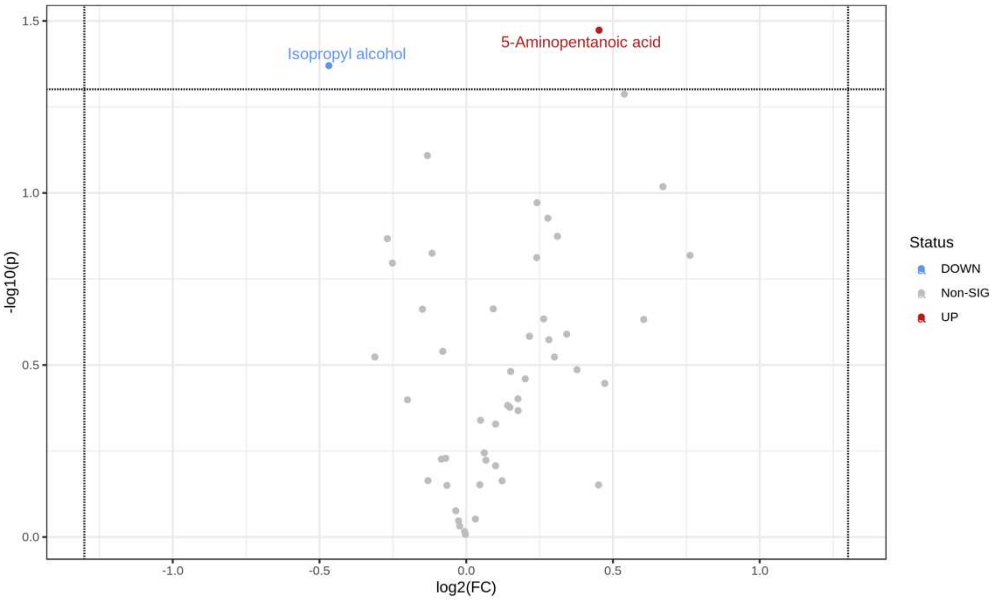

Figure S2. Volcano plot: pairwise comparison placebo vs. probiotics time point t_0 in stool. The $\log_2$ fold change (log2 FC) (x-axis) is plotted against the corresponding adjusted p-value (y-axis). Thresholds for significance (adjusted p-value = 0.05; horizontal dashed line) and for changes in concentration, vertical dashed lines are shown. Significant increased metabolites compared to placebo at t_0 are displayed as dots in dark red on the right side, respectively. Significant decreased metabolites compared to placebo at t_0 are displayed as light blue dots on the left side, respectively. Non significantly altered metabolites are shown as grey dots.

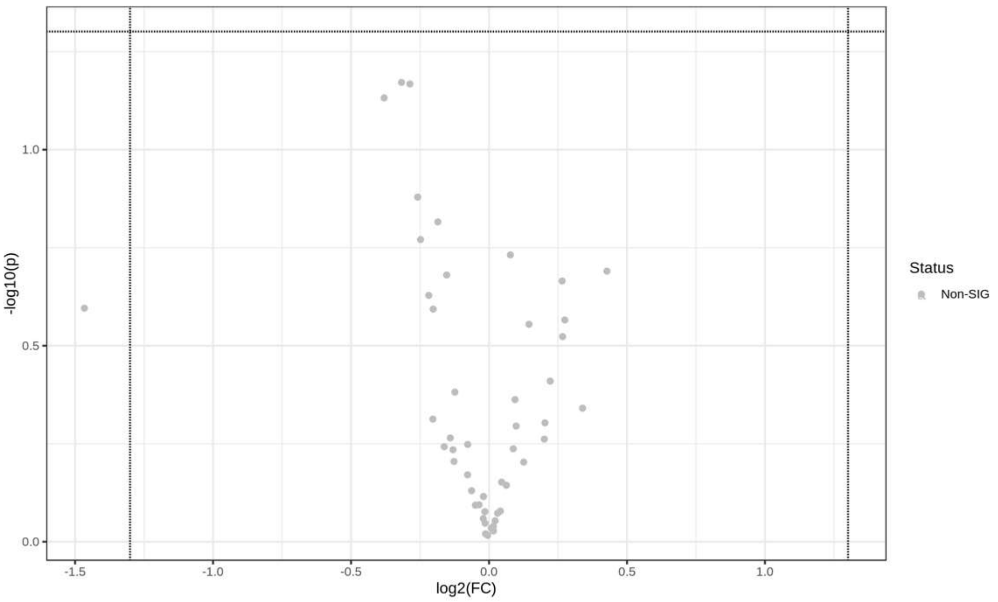

Figure S3. Volcano plot. Placebo group longitudinal pairwise comparison of time point t_0 and t_2 in stool. The log2 fold change (log2 FC) (x-axis) is plotted against the corresponding adjusted p-value (y-axis). Thresholds for significance (adjusted p-value = 0.05; horizontal dashed line) and for changes in concentration, vertical dashed lines are shown. Not significantly altered metabolites are shown as grey dots.

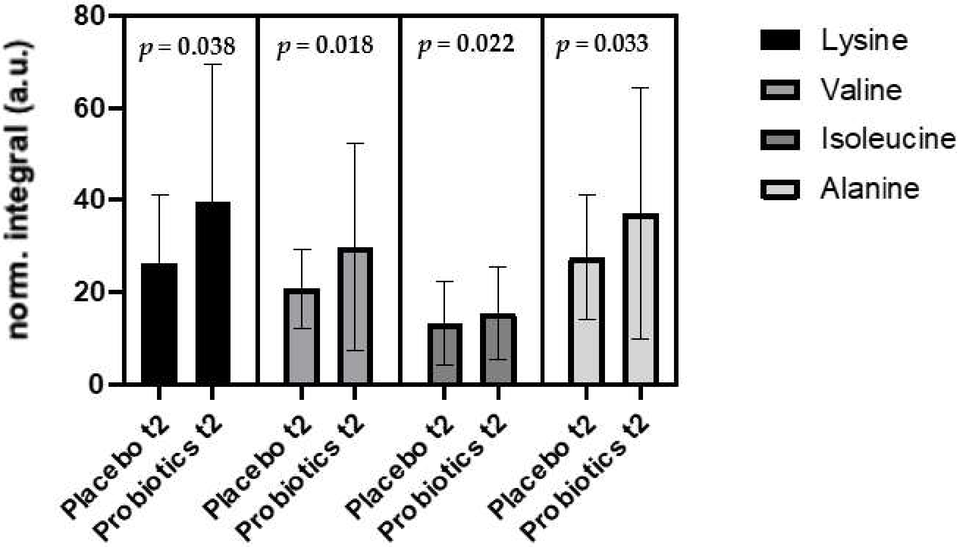

Figure S4. Boxplots univariate analysis of most pronounced metabolites in stool**.** Comparison between placebo (n = 29) and probiotics group (n = 28) after 28-days.

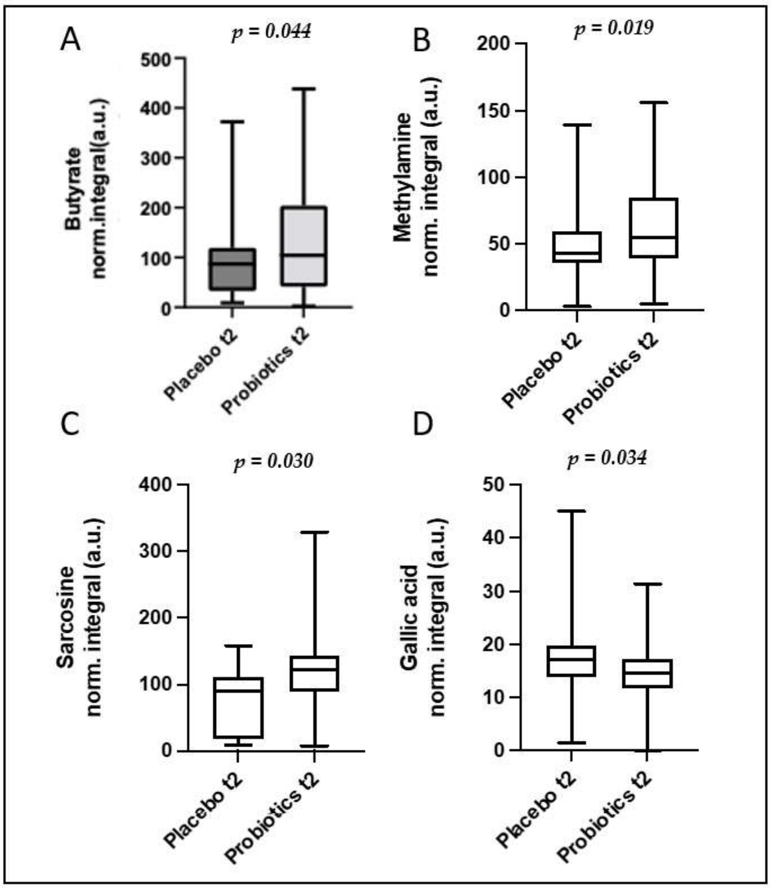

Figure S5. Boxplots univariate analysis of most pronounced metabolites in stool. Comparison between Placebo (n = 29) and probiotics group (n = 28) after 28-days (A) Butyrate ($p = 0.044$), (B) Methylamine ($p = 0.019$), (C) Sarcosine ($p = 0.034$); Gallic acid ($p = 0.030$).

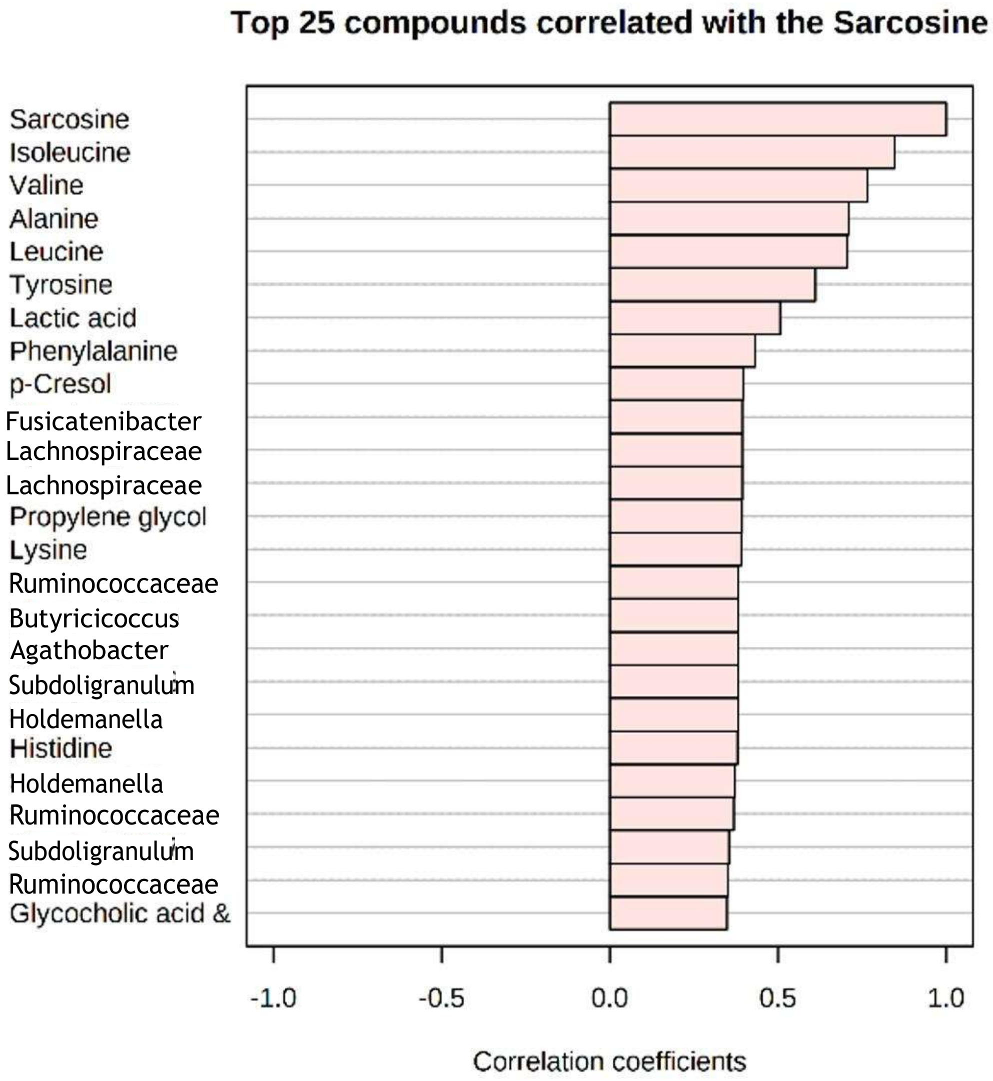

Figure S6. Top 25 Correlations coefficients with the normalized concentrations of Sarcosine between other metabolites and the relative abundance of bacterial species in 16S rRNA sequencing

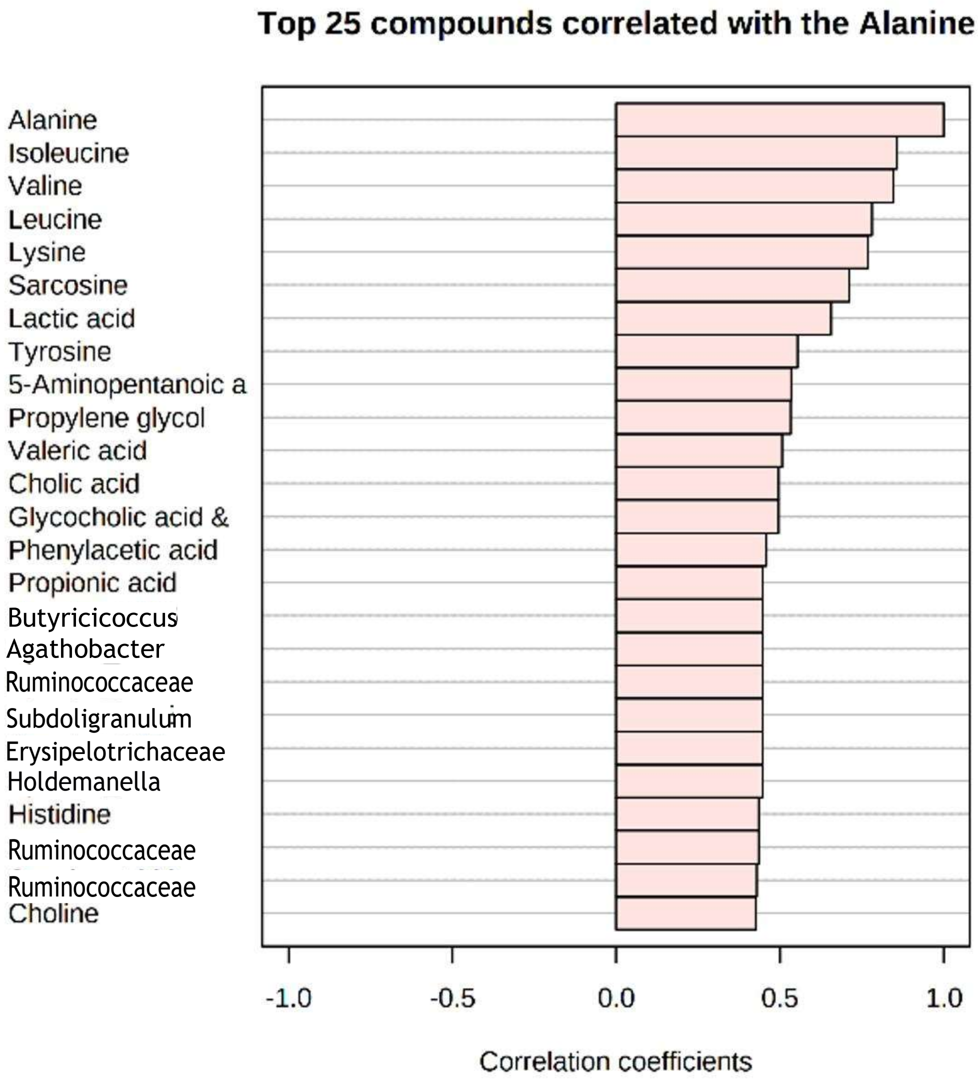

Figure S7. Top 25 Correlations coefficients with the normalized concentrations of Alanine between other metabolites and the relative abundance of bacterial species in 16S rRNA sequencing

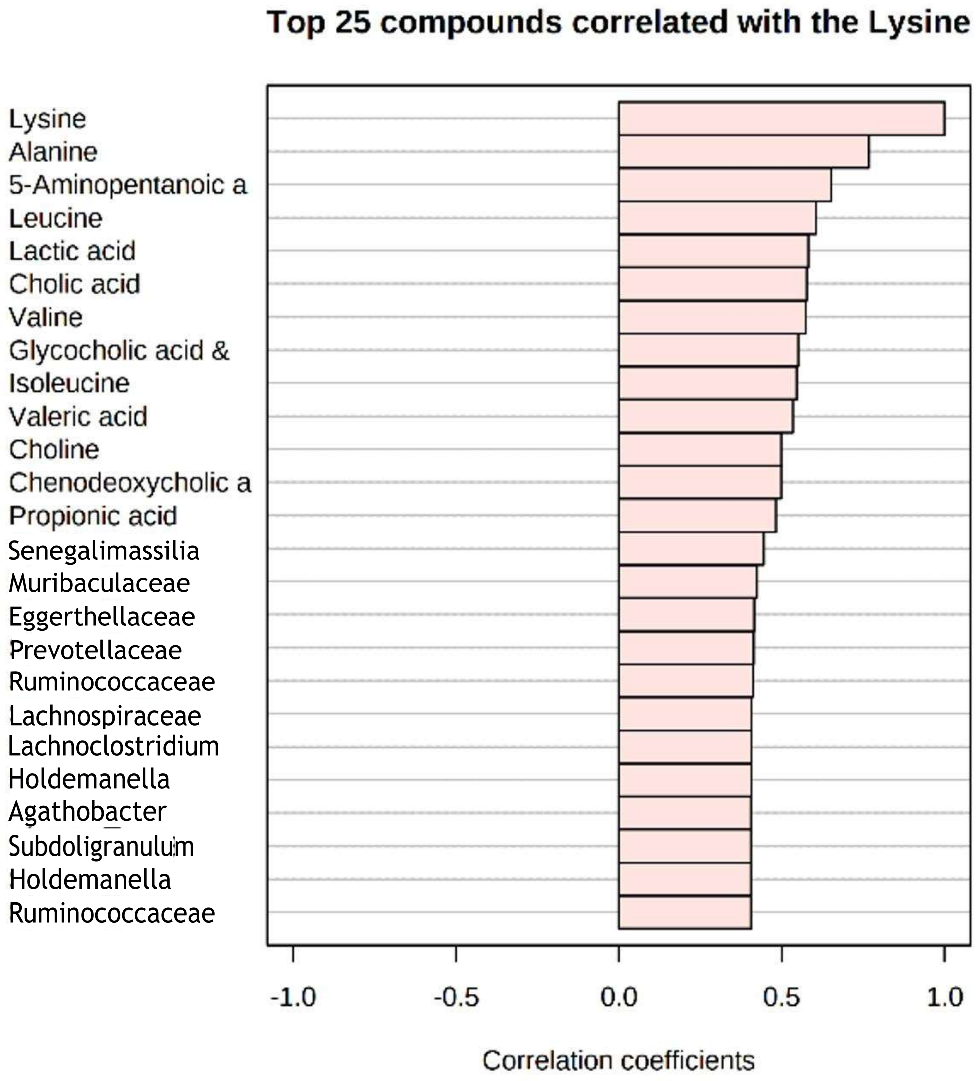

Figure S8. Top 25 Correlations coefficients with the normalized concentrations of Lysine between other metabolites and the relative abundance of bacterial species in 16S rRNA sequencing

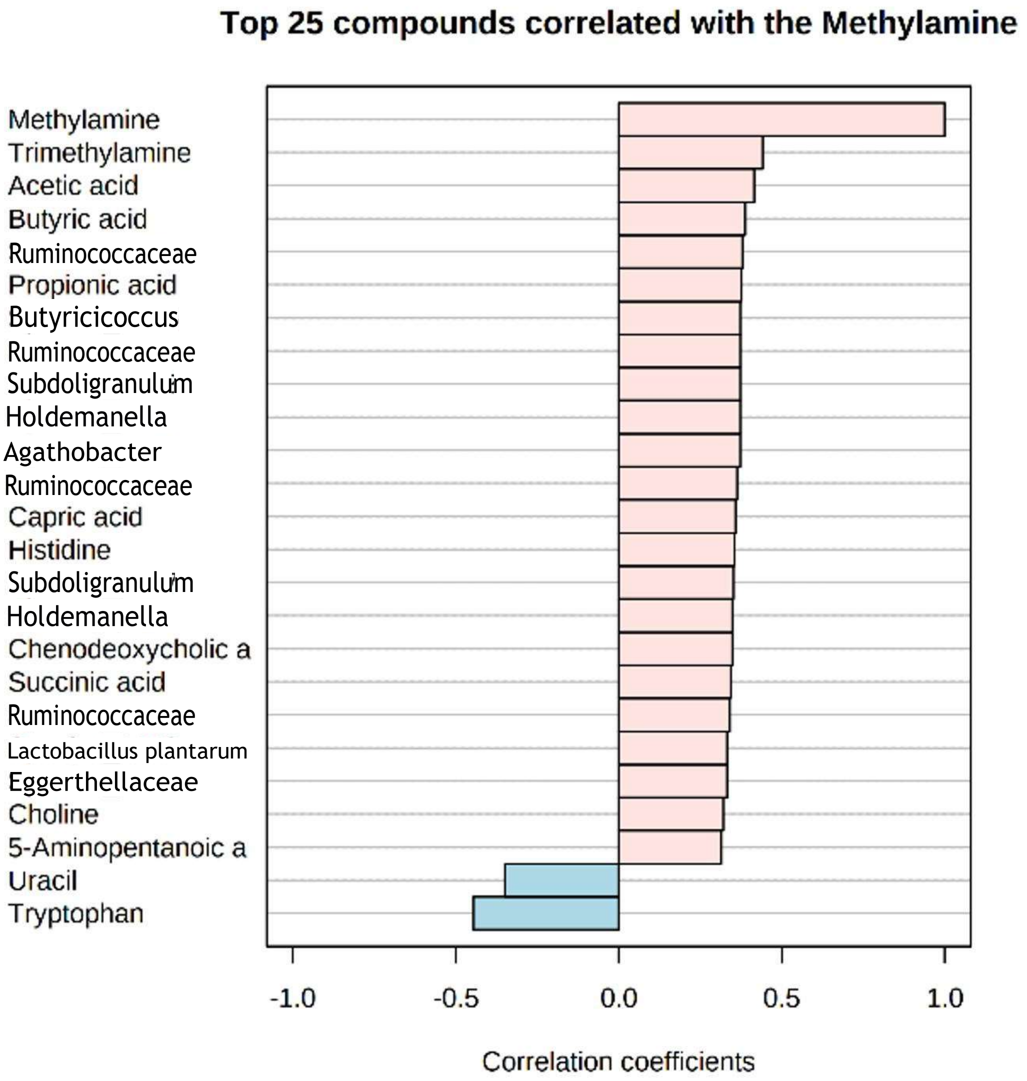

Figure S9. Top 25 Correlations coefficients with the normalized concentrations of Methylamine between other metabolites and the relative abundance of bacterial species in 16S rRNA sequencing

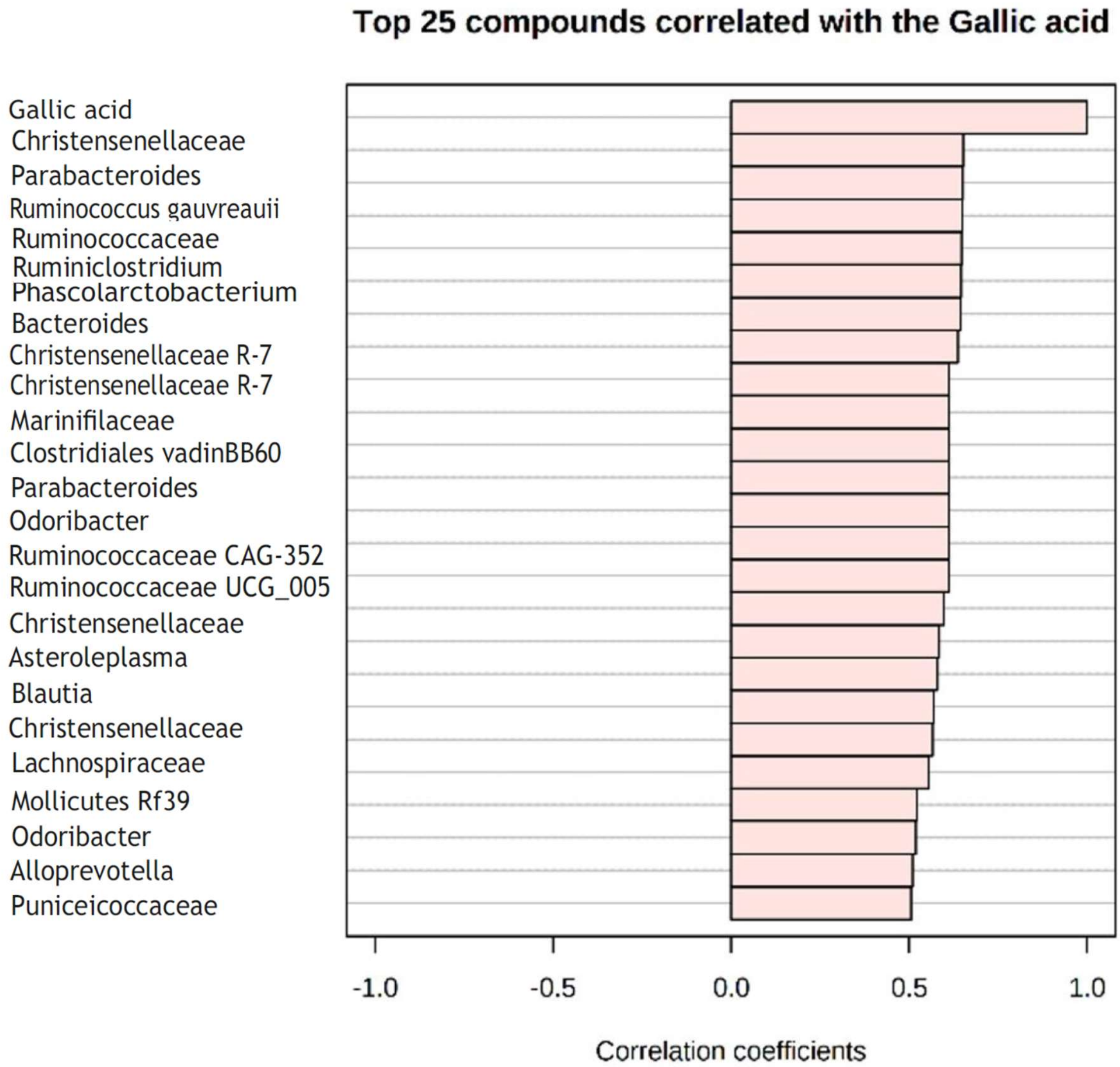

Figure S10. Top 25 Correlations coefficients with the normalized concentrations of Gallic acid between other metabolites and the relative abundance of bacterial species in 16S rRNA sequencing

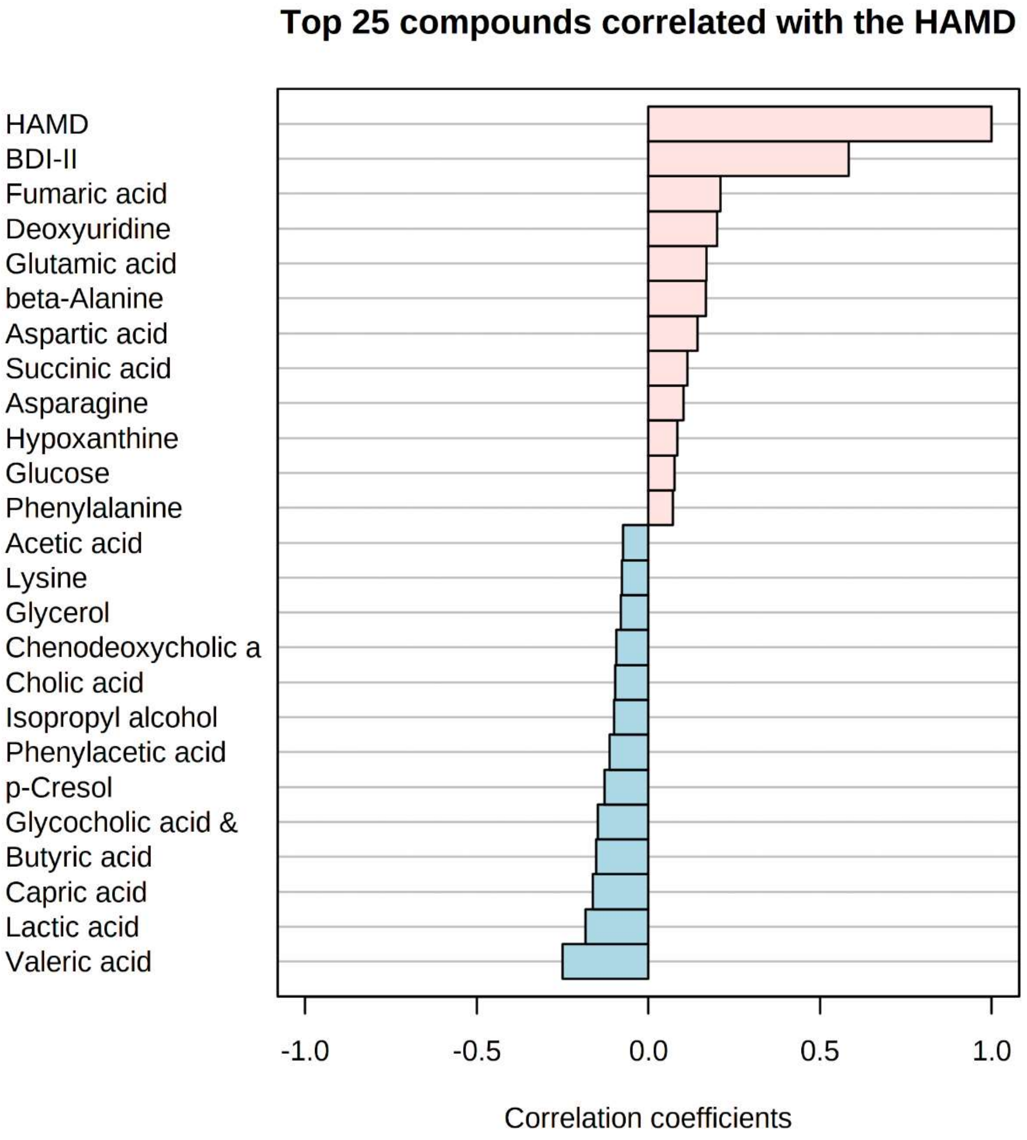

Figure S11. Top 25 Correlations coefficients of metabolites (normalized concentrations) with the HAMD.

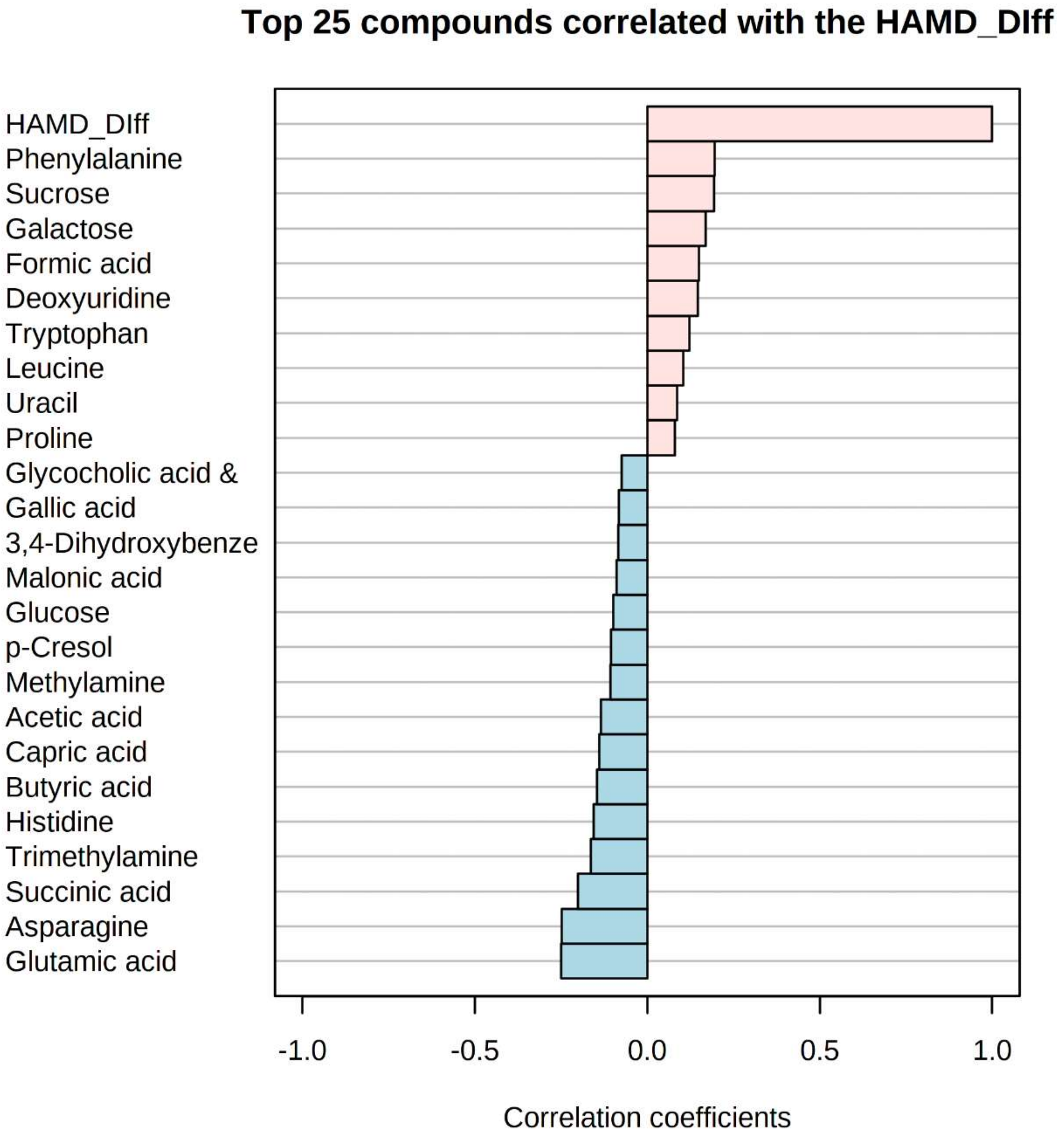

Figure S12. Top 25 Correlations coefficients of metabolites (normalized concentrations) with the HAMD difference between baseline and intervention samples.

Table S1. Significant metabolic changes in stool, determined by untargeted ^1H NMR spectroscopy.

Metabolites	Probiotics (N = 28)	Placebo (N = 29)	*p*-value
	**Median (25th – 75th percentile)**	**Median (25th – 75th percentile)**	*Sig.*
Butyric acid	127.82 (52.28 - 204.98)	93.55 (54.93 - 123.56)	0.044
Lysine	34.20 s (26.85 - 52.31)	29.99 (24.09 - 33.75)	0.038
Alanine	34.37 (28.47 - 41.47)	31.02 (21.99 - 40.42)	0.033
Sarcosine	12.29 (9.25 - 14.14)	10.83 (8.98 - 13.5)	0.030
Isoleucine	14.21 (9.79 - 19.26)	10.72 (9.16 - 15.71)	0.022
Methylamine	5.46 (4.11 - 7.57)	4.33 (4.12 - 5.51)	0.019
Valine	26.71 (19.15 - 33,75)	23,70 (17.66 - 28.39)	0.018
Gallic acid	1,58 (1.26 - 1.82)	1.71 (1.39 - 1.96)	0.034

Note: Sig. = Significance, results are the values of normalized integral (a.u.)
